# Comparison of corneal endothelial cell measurements by two non-contact specular microscopes

**DOI:** 10.1186/s12886-015-0068-1

**Published:** 2015-07-29

**Authors:** Laura Gasser, Thomas Reinhard, Daniel Böhringer

**Affiliations:** Eye Center, University Hospital Freiburg, Killianstr. 5, Freiburg, 79106 Germany

**Keywords:** Cornea, Endothelium, Cell density, Specular microscope, Comparison

## Abstract

**Background:**

Measurement of corneal endothelial cell density is important both for clinical diagnosis as well as clinical studies. Since endothelial cell loss is considered irreversible in humans, even small changes in endothelial cell density are relevant. Therefore it is important to know whether different instruments for endothelial cell density measurements give the same results and can thus be used interchangeably. In this study we compare corneal endothelial cell density and morphometry measurements from two widely used non-contact specular microscopes, the Topcon SP3000P and Konan Noncon Robo SP8000.

**Methods:**

Endothelial cell measurements were performed with both the Topcon SP3000P and Konan Noncon Robo SP8000 on 34 eyes of 18 consecutive patients of our cornea clinics with poor image quality being the only exclusion criterion. Images were obtained using the auto-focussing method and manual cell selection. Endothelial cell density (ECD), hexagonal cell ratio (HEX) and coefficient of value (CV) of the endothelial cell layer were calculated by the instruments’ built-in software.

**Results:**

ECD values calculated by the Konan were systematically higher than Topcon values: in 94 % of eyes Konan gave a higher value than Topcon, leading to a mean difference in ECD between the instruments of 187 cells/mm^2^ (*P* < 0.001 in paired Wilcoxon test). HEX showed a broad range of values and differed greatly with only weak correlation between the two instruments. CV values for Konan mostly exceeded Topcon values, and only showed a weak correlation between the two instruments as well.

**Conclusions:**

Values for ECD between the Konan and the Topcon do correlate well, but the ECDs calculated by the Konan are systematically higher than Topcon values. Both HEX and CV vary greatly and do not correlate sufficiently. Thus we recommend not to use the Konan and the Topcon instrument interchangeably.

## Background

Specular microscopy of the corneal endothelial cell layer is an important diagnostic tool in clinical practice [1, 2]. It is not only used to assess the health of the endothelium in patients with corneal diseases, but is also part of the routine examinations after corneal transplantation. In addition to its clinical use, follow-up endothelial cell measurements are used in clinical trials to assess the corneal safety of surgical techniques or new materials. Endothelial cell measurements are often repeated over time to analyze changes in the endothelial cell layer. However, the microscopes used in one clinic might be changed as time passes. Alternatively, differing microscopes may be in use in different trial sites. It is therefore important to know whether different models of specular microscopes give the same results and can be used interchangeably.

The Konan Noncon Robo SP8000 and the Topcon SP3000P are two modern autofocussing specular microscopes which analyze the central corneal endothelium. They are among the most widely used non-contact specular microscopes, and were thus compared in this study regarding their values for endothelial cell density and cell morphometry parameters.

## Methods

### Patient selection

Endothelial cell measurements were performed with both Topcon SP3000P and Konan Noncon Robo SP8000, two widely-used non-contact specular microscopes. We examined 34 eyes of 18 patients of our cornea clinic: All consecutive patients that received an endothelial cell density measurement for diagnostic reasons independent of the underlying condition were included in the analysis to obtain a wide range of endothelial cell densities and morphologies. Only eyes with poor image quality were excluded from this study. All patients consented for endothelial cell measurement acquisition. Further analysis of cell counts were performed anonymously. The study has been approved by the ethics committee of the University of Freiburg, Germany.

### Image acquisition

The measurements with the two different instruments, the Topcon SP3000P (Topcon Corporation, Tokyo, Japan) and the Konan Noncon Robo SP8000 (Konan Medical, Hyogo, Japan), were performed by the same technician and on the same day. Patients were asked always to look at the central fixation target. For both instruments, the auto-alignment function was used.

**Fig 1:**
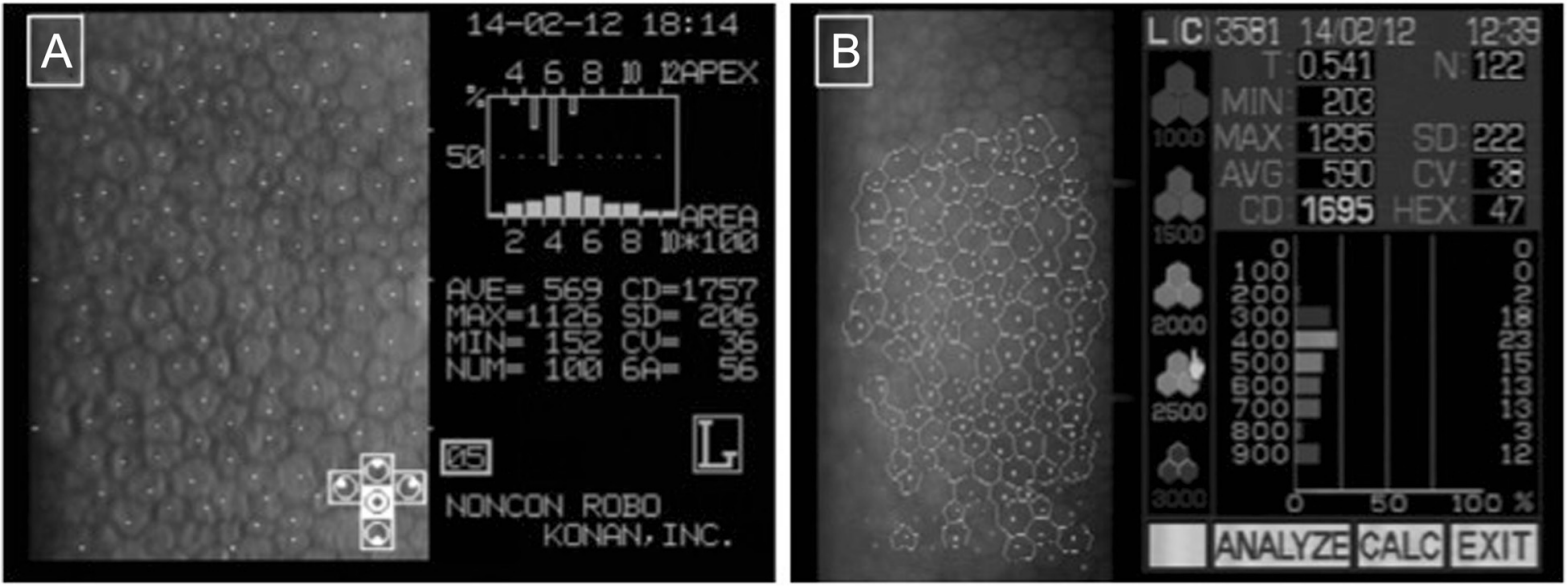
Representative endothelial cell measurements by the two instruments of the same patient. **a**: Konan measurement. (“CD”: Endothelial cell density, “CV”: coefficient of value, “6A”: hexagonal cell ratio, “NUM”: Number of cells included in the analysis). **b**: Topcon measurement. (“CD”: Endothelial cell density, “CV”: coefficient of value, “HEX”: hexagonal cell ratio, “N”: Number of cells included in the analysis)

For the Konan instrument, all corneal endothelial cells which were clearly visible on the picture were marked manually; for the Topcon microscope, as many of the clearly visible endothelial cells as allowed by the built-in software were marked manually. Endothelial cell density (ECD), hexagonal cell ratio (HEX) and coefficient of value (CV) of the endothelial cell layer were calculated by the instruments’ built-in software (see Fig. 1).

### Statistical analysis

A comparison of ECD, HEX and CV of the endothelial cell layer between the two instruments was performed using the non-parametric paired Wilcoxon test and Spearman correlation.

Results are shown as Bland-Altman analysis [3]. Statistical analyses were carried out with “R” [4]. *P* < 0.05 was considered as statistically significant.

## Results

### Patient demographics

Patient age ranged from 23 to 82 years with a median of 45 years. 56 % of patients and eyes were female.

For the Konan 110 ± 45 cells/measurement depending on the cell density were included in each analysis, and for the Topcon 100 ± 30 cells/measurements. This difference was not statistically significant (*p* = 0.16).

Including both treated and untreated patients, a wide range of endothelial cell densities from around 600 to 3000/mm^2^ (580–2869 cells/mm^2^ according to Topcon, or 676–3174 cells/mm^2^ according to Konan, respectively) was analyzed in this study. Around one third of eyes had an endothelial cell density below 2000/mm^2^.

### Endothelial cell density (ECD) (Fig. 2)

**Fig 2:**
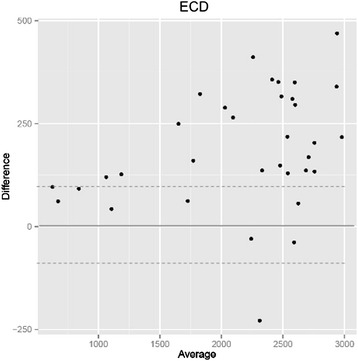
Systematically higher endothelial cell densities (ECD) with the Konan specular microscope compared to the Topcon specular microscope: The ECD was measured in the same eyes on the same day with two instruments. Even though the values between the two instruments correlate with each other, 94 % of measurements with the Konan specular microscope showed higher ECDs than the corresponding measurement with the Topcon microscope (*p* < 0.001 in paired Wilcoxon test). The Bland-Altman-analysis shows that 74 % (26 of 34) of endothelial cell density measurements differ by more than 100 cells/mm^2^ (broken lines)

The endothelial cell density measurements differed statistically significant between the two instruments (*p* < 0.001): The cell densities calculated by the Konan were higher than the Topcon values in 32 out of 34 (94 %) of eyes with a mean of 187 cells/mm^2^ (−228 to 470/mm^2^) difference. The mean ECD was 2252 ± 704/ mm^2^ for Konan and 2065 ± 657/mm^2^ for Topcon, respectively. The difference from the Topcon ECD to the Konan values was −1 to +20 % with a mean deviation of +9.6 %. The strongest deviation between the two measurements was 470/mm^2^ or 20 % of the Topcon ECD.

We found higher values for the Konan compared to Topcon throughout the cohort (range of ECD from around 600-3000/mm^2^), but observed a trend for stronger deviations for higher cell densities. Spearman correlation for ECD between the instruments showed a good correlation of rho = 0.94 (*p* < 0.01). Thus the ECD measured with the two instruments correlated with each other, but the Konan ECDs were statistically significantly higher than the Topcon ECDs.

### Hexagonal cell ratio (HEX) (Fig. 3)

**Fig. 3:**
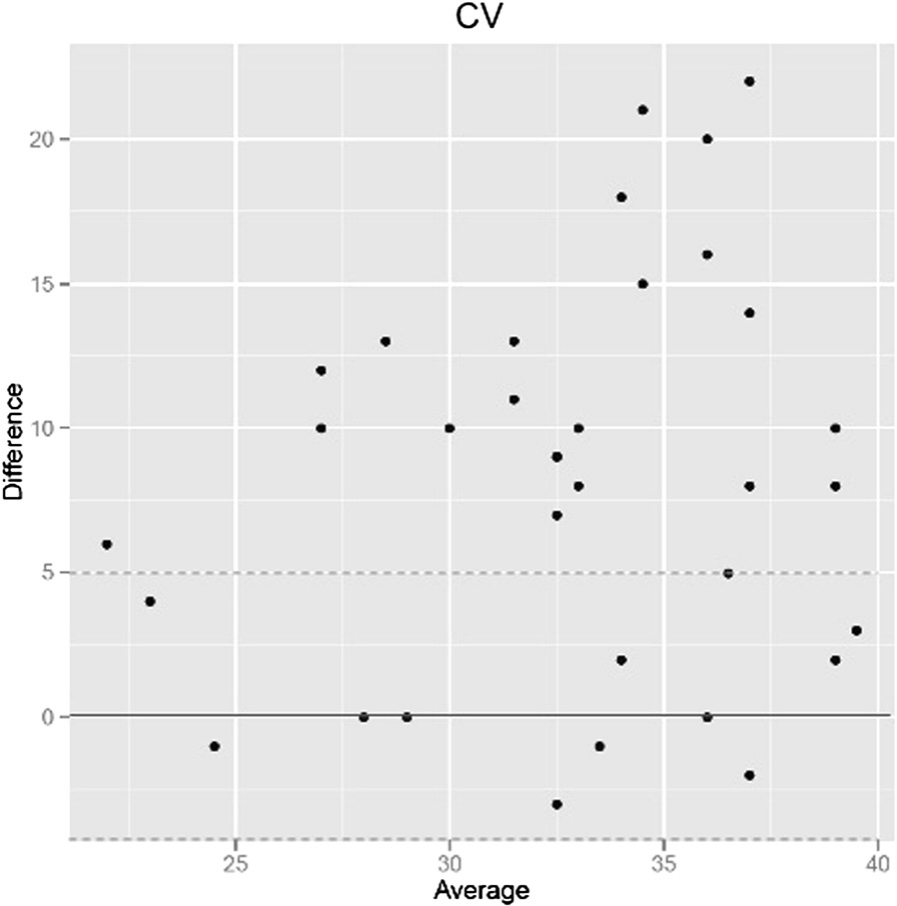
Poor correlation between hexagonal cell ratio (HEX) of endothelial cell measurements with the Topcon and the Konan microscope: Bland-Altman-analysis. 62 % (21 of 34) of hexagonal cell ratio measurements with the two instruments show a greater than five percent difference (broken lines), and 24 % of measurements (8 of 34) differ by more than ten percent

The rate of hexagonal endothelial cells is used as a marker for cell polymorphia. The values given by the two instruments vary greatly and show only a weak correlation. For the Konan, hexagonality values ranged from 45–70 (mean 56) and for the Topcon, values of 0–100 (mean 55) were given (*p* = 0.82). The differences of values between the two instruments ranged from −45 to +60.

### Coefficient of value (CV) (Fig. 4)

**Fig. 4:**
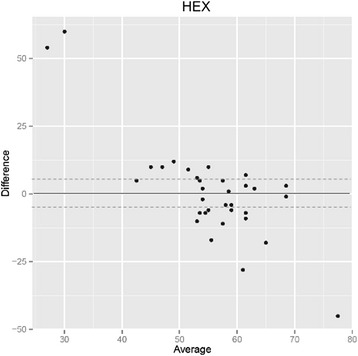
Poor correlation between coefficient of value (CV) of endothelial cell measurements with the Topcon and the Konan microscope: Bland-Altman-analysis. The CV values between the two instruments show a greater than five percent difference (broken lines) in 65 % of measurements (22 of 34), and a greater than ten percent difference in 32 % of measurements (11 of 34)

The coefficients of value given by the two microscopes differ greatly and show only a weak correlation. In general, mean values were statistically significantly higher for the Konan with 37 ± 6 versus 29 ± 5 for the Topcon (*p* < 0.001). The values for the two instruments differed from −3 to 22.

## Discussion

Endothelial cell density and morphometry are essential for adequate follow-up of corneal grafts and diseases. Fast, easy-to-use and reliable instruments to measure the endothelial cell layer are required in clinical routine. While contact instruments provide excellent images, these have the disadvantage of directly touching the cornea. Non-contact specular microscopes are appreciated by clinicians and patients for their convenient handling. In this study we focussed on two non-contact instruments from Konan and Topcon, two widely used non-contact specular microscope manufactors in this field. 

The models used in this study are the Konan Noncon Robo SP8000 from the Konan Robo SP series and the Topcon SP3000P from the Topcon SP-series, which provide high magnification views of specular reflected light from the corneal endothelium. Both offer auto-alignment to capture the images. Several methods of cell analysis can be applied: Manual, semi-automatic or automatic cell counting strategies. While automatic strategies are appealing because of ease of use, these are known to be less accurate than semi-manual or manual cell detection [5]. Thus we used manual cell detection for both instruments in the present study.

In addition, the number of marked cells that are incorporated into the cell density calculation can influence the results for cell density [6–8]. It is advised to dot as many cells as possible, since the more cells are included in the analysis the smaller the resulting variations [9]. In the present study, we therefore marked and thus included into the calculations as many cells as possible for the two devices: All clearly visible cells on the picture for the Konan Robo instrument were selected, and for the Topcon device, cells were manually marked until the built-in software ceased further cell marking. These methods resulted in a tendency for a higher number of analyzed cells for the Topcon instrument without statistical significance.

As it is also known that inter-observer variation can occur, all examinations and cell dotting with both instruments were performed by the same examiner.

Other study limitations are the cohort size. As the performance of measuring instruments und thus the congruence of two machines might be dependent on cell density and regularity, we included not only healthy, untreated corneas but a wide range of patients to include also a wide range of endothelial cell mosaics into our comparison. The analysis covers endothelial cell counts from as low as about 600 cells/mm^2^ to around 3000 cells/mm^2^, with around one third of measurements below 2000 cells/mm^2^. According to a linear regression model to declare influencing factors on the measurement agreement for the two instruments, neither a low ECD nor age turned out statistically significant predictors of the difference between endothelial cell measurements of both instruments. Nonetheless, it is possible that in special situations like e.g. certain diseases as cornea guttata, or poor image quality (which was not included in our study) the observed difference between the instruments might be altered. However, even with a limited number of eyes examined, and a wide range of cell densities taken into account, our study shows that results are rather consistent: In 94 % of examinations, Konan gave higher cell counts for ECD than Topcon.

Since ECD measurements are mostly used for follow-up of endothelial cell changes in individual patients or for clinical studies, it is important to know if or which instruments can be used interchangeably. Several studies have compared endothelial cell counts from models of the Topcon SP series with other contact [10] or non-contact microscopes [11, 12]. Thuret et al. compared the Topcon SP2000P with a non-contact specular microscope from Rhine-Tec, Germany. When using the semi-automatic mode for both instruments, agreement was far better than compared to the automatic mode, but still the Rhine-Tec showed the tendency to overestimate low and underestimate high endothelial cell densities compared to the Topcon [12]. De Sanctis et al. compared the same Topcon instrument SP2000P with the Konan CC7000 non-contact specular microscope. In this analysis, the endothelial cell densities measured by the Konan where statistically significant higher than those calculated by the Topcon; depending on the examiner, the mean difference in ECD was 185 to 229 cells between the two instruments [11]. In our analysis, we used different models, but instruments from the same manufactorers, and found similar results: We found higher ECD for our Konan model compared to the Topcon instrument. However, we also included patients with corneal disease or following keratoplasty to analyze a wider range of ECDs, while in the study by de Sanctis et al. only untreated healthy subjects where enrolled. In addition, they used the semi-automatic mode and marked 75–88 or 80 cells, respectively, while we used the manual mode and marked a mean of 100 or 110 cells respectively per image to minimize discrepancies caused by calculation inaccuracies due to low numbers of included cells. Apart from the mentioned study, Konan non-contact microscopes have also been compared to contact microscopes for ECD measurements [13, 14]. When comparing the Konan Robo SP8000 to a non-contact instrument by Zeiss regarding mean cell area, significant differences where detected so that the authors recommend not to use the instruments interchangeably [13]. Several studies have shown that results for ECD, hexagonality or cell polymorphism can differ significantly. Luft et al. compared a Konan instrument (CellChek XL) to three non-contact models from other manufacturers (Bon Optics, Tomey and Nidek) both in healthy and compromised corneas. They found little consistency between the 4 devices with respect to the qualitative endothelial cell parameters CV and hexagonality readings [15].

Due to the discrepancies in all parameters tested in our own study, we recommend not to use the Konan and the Topcon interchangeably in the same patient.

## Conclusions

The current study reminds us that different non-contact specular microscopes for endothelial cell measurements are not readily comparable. When a switch to a new model of endothelial cell microscope is inevitable in clinical routine, we suggest an overlap with both instruments during which patients should be examined by both the old and the new instrument at the same visit to better adapt the follow-up values for the patients. When carrying out clinical studies involving several study centers with different microscopes, we suggest endothelial cell measurements should be analyzed by a central reading center.

## Abbreviations

ECD: Endothelial cell density
HEX: Hexagonal cell ratio
CV: Coefficient of value
